# Dr. Kinglake, on Mr. O'Neal's Case

**Published:** 1805-03-01

**Authors:** Robert Kinglake

**Affiliations:** Taunton


### *To the Editors of the Medical and Physical Journal.*

Gentlemen,

Accept my respectful acknowledgment of your liberality, in favoring me with an opportunity of remarking on Mr. O'Neal's case, by sending me a copy of it previously to its being published.

The case is undoubtedly a very interesting one, but does not appear to me to involve the credit of the cooling treatment of gout. It should seem, that an unyielding affection of the stomach prevailed from the commencement to the termination of the disease. It is not stated that the gouty tumefaction on the foot alleviated the stomachic complaint; nor is any thing said either as to the state of the pulse, or to that of the motive condition of the nervous system.

Topical cold, as usual, removed the local inflammation, but it does not appear that the stomach suffered an aggravated degree of affection from its removal; on the contrary, less subsequent complaint was made of that organ, and sound sleep ensued.

With respect to the true nature of the stomachic affection, Mr. O'Neal is most competent to form a correct judgment of it, from his opportunity of minutely observing the existing circumstances. A sense of coldness is surely not a symptom of gouty inflammation; it rather indicates deficient than augmented excitement.

The external application of cold water to the region of the stomach is only vindicable by Mr. O'Neal's circumstantial acquaintance with the case. How far such an attempt to relieve might have succeeded in reducing positive inflammation is not in my power to determine, having never carried topical cold to that length of experiment, tho' it would seem to be an appropriate remedy.

The symptoms which more particularly indicate stomachic inflammation, are a sense of burning pain in the cavity of that organ, extreme dryness of tongue, insatiable thirst, and a rapid hard pulse. Instead of these denotations of excessive temperature, an inveterate feeling of coldness in the region of the stomach seemed to have predominated in the case in question, which gradually involved the brain in a state of comatose stupor. All this evinced failure of nervous energy, and certainly constituted the usual character of apoplectic malady.

Mr. O'Neal, from a much more accurate knowledge of the subject, than is possible for me to learn from a general statement, thinks the complaint was from its commencement of an apoplectic tendency, and that its fatal termination could not have been obviated. This was probably the true state of the case.

Mr. O'Neal has, with a laudable spirit of inquiry, subjected the cooling treatment of gout to trial, and he will have no reason to regret his persuasion of the salutary efficacy of the practice, if he should correctly direct the course of his experiments by the indications of sensual temperature.

It behoves me to suggest to Mr. O'Neal and to the public at large, that the cooling treatment of gout was never proposed on my authority to be extended to the regions of the visceral organs by topical affusion with cold water. It is an essential part of my doctrine that gout cannot occur on the visceral parts, for want of the requisite structure to give effect to that peculiar inflammation. These parts might, and often actually are, seriously disordered by the sympathetic influence of protracted gouty pain on the extremities; but it is for me yet to learn that the cessation of torture on these parts, can give rise to fatal excitement, whether inflammatory or nervous, on the visceral organs.

The cooling treatment of gout, like other modes of remedy, must necessarily be liable to various morbid coincidences of the system, but such accidental occurrences should stand upon their own ground; if inflammatory, they should be appropriately treated; if spasmodic, managed accordingly. Thus, if during the refrigerant treatment of gout, a sense of coldness at the stomach should occur, it would be opposing the sensual indication of cure to increase that coldness; genial warmth should be applied, by moderately exciting the stomach either dietetically or medicinally.

If spasmodic twitches should arise, opium and other suitable narcotics should be duly given. So likewise, if the head be affected, it should be treated according to the degree and kind of morbid action. When tone, energy, and excessive heat abound, diminished temperature will afford relief; but if tremor, debility, and coldness prevail, additional excitement will be indicated.

Much diseased sympathy subsists between the similar structure of the different joints; a reciprocal interchange therefore of gouty excitement between these kindred parts is very apt to obtain, and does often prevail with inveterate obstinacy, obstinacy, but without at all involving the safety of the general health. In these instances no quarter should be given the shifting stations. The new excitement is always unequivocally announced by inflammatory heat and pain, which constitute the essential character of gout.

The visceral parts are also liable to inflammation, and were they susceptible of being affected by it by gouty transference from the joints, it would not be denoted by a failure of nervous energy, evinced by coldness of stomach, and stupor of brain, but by arterial throbbing, burning heat, exquisite torture, and violent delirium.

Under my own direction the cooling treatment of gout has succeeded without having been embarrassed by even an accidental occurrence of visceral discomposure. When irresolution or dread of any noxious effect of the treatment has induced any thing like a tremulous state of excitability, it has been my uniform practice (as mentioned in my Dissertation on Gout) to give from one to four tea spoons full of equal parts of camphorated tincture of opium and ammoniated tincture of guaiacum, in any suitable vehicle. This compound medicine has a tendency to calm morbid disturbance, and might salutarily obviate its hurtful concentration on any particular viscus.

The bold and fearless freely use the remedy without either advice or precaution, and have in no instance to my knowledge, suffered from their dauntless trial of its efficacy.

The cooling treatment of gout is ushered into the world on the firm basis of correct experience; if that support will not entitle it to an impartial scrutiny, and a fair estimate of its merits, it shall receive from me no speculative recommendation. Imaginary cases will often occur in opposition to its credit; but these, when stripped of the blandishments of prejudice, error, and fiction, will lose much of their seeming validity.

The public are put on their guard in a caution suggested by me respecting the various artifices that already have, and probably will continue to be, practised against the credit of the treatment, in my Reply just published to Mr. Edlin's two reputed cases of its fatal effects, to which the reader is referred for a view of the grounds on which the refutation of those cases is attempted, and for other information relating to the refrigerant treatment of gout.

It is my only wish in this arduous inquiry to ascertain the truth of the question; the research was instituted with an heartfelt regard to the benefit of mankind, and whether it it be accomplished by the eventual establishment or rejection of my proposal, the philanthropy of my object will be equally gratified. I am, &c.

*Taunton, Dec.* 14, 1804. ROBERT KINGLAKE.

